# Volatiles in the mantle transition zone and their effects on big mantle wedge systems

**DOI:** 10.1093/nsr/nwae136

**Published:** 2024-04-04

**Authors:** Yu Wang, Yi-Gang Xu

**Affiliations:** State Key Laboratory of Isotope Geochemistry, Guangzhou Institute of Geochemistry, Chinese Academy of Sciences, China; State Key Laboratory of Isotope Geochemistry, Guangzhou Institute of Geochemistry, Chinese Academy of Sciences, China; Southern Marine Science and Engineering Guangdong Laboratory (Guangzhou), China

The mantle transition zone (MTZ), located between the upper and lower mantle, is characterized by sudden seismic-velocity discontinuities at depths of 410 and 660 km where significant change in mineralogical composition occurs. As a link between the upper and lower mantle, the MTZ is a layer of special significance in Earth's deep structure, and a critical interface in geodynamics and material circulation. Moreover, it has been increasingly recognized as a potential considerable repository for volatiles, and exerts great influence on the mantle dynamics, melting processes, diamond formation, volcanic activity and volatile cycling of the Earth. For example, the MTZ may represent the largest water reservoir hidden in the deep Earth, given the high water-storage capacity of its minerals, which could induce extensive dehydration melting of vertically flowing mantle, fundamentally changing our perspective on global water circulation and mantle properties [1]. An MTZ water-filter model has been proposed to account for many of the observations of geochemical reservoirs, e.g. oceanic island basalt (OIB) and mid-ocean ridge basalt (MORB) source regions, and early production of enriched continental crust without invoking layered convection and all its attendant problems [2]. Therefore, the MTZ has the potential to engender a seminal paradigm shift in terms of volatile cycling, serving as a pivotal catalyst and a crucial driver for advancing deep Earth science. In this perspective, we review the progress and existing challenges of this significant frontier, presenting key focal points that will require dedicated attention in the future.

Many geophysical observations such as electrical conductivity and seismic velocities (and attenuation) suggest that the MTZ could be hydrous. Furthermore, experiments show that high-pressure polymorphs of olivine (wadsleyite and ringwoodite), majoritic garnet and many hydrous phases (phase B, C, D, Egg etc.) have the capacity to accommodate H_2_O. Nonetheless, scientists were skeptical about the presence of water in the MTZ until Pearson *et al.* [3] reported the first evidence for a water-rich ringwoodite inclusion in a natural diamond from Juína, Brazil, which could host ∼1.4 wt% of H_2_O. They estimate that, at least locally, the MTZ is hydrous to ∼1 wt%. However, the debate pertaining to whether the MTZ is dry or wet remains, and the estimated water content of the MTZ varies from ∼2.5 surface ocean volumes to as little as 0.03 wt%, and even those in favor of a wet MTZ are often challenged by geophysical observations that the MTZ is only locally hydrated.

**Figure 1.**
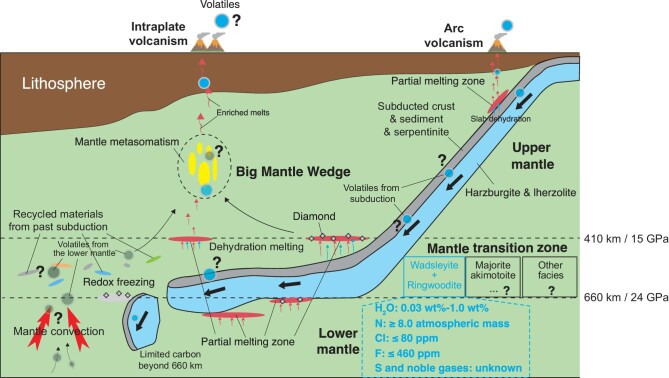
Schematic cartoon illustrating the significance of the mantle transition zone (MTZ) and the big mantle wedge (BMW) system in governing volatile cycling of the Earth. The materials and associated volatiles in the MTZ are either introduced by slab subduction and stagnation or recycled from the lower mantle by large-scale mantle convection. Estimated budgets of various volatiles in the MTZ and their fluxes from the MTZ to BMW are subject to large uncertainties, due to limited knowledge of the behaviors of volatiles and degassing during subduction from shallow depths to the transition zone, and during recycling from the lower mantle to the transition zone.

One of the widely acknowledged locations where water is believed to be present in the MTZ is eastern Asia [4], a vast area characterized by the existence of stagnant subducted slab in the MTZ detected by seismic tomography [5]. As the presence of water significantly lowers the solidus of mantle rocks and slab materials, the partial melting of peridotite or a stagnant slab in the transition zone could release appreciable amounts of volatiles into the big mantle wedge (BMW), the upper mantle part above the stagnant slab. Subsequent volatile-assisted melting within the BMW eventually leads to the formation of intraplate magmatism [6,7]. In other words, aside from the thermal structure of the slab, the melting process in the MTZ critically depends on whether the MTZ is wet and how much water is in there. A minimum of 0.05 wt% H_2_O was estimated to cause partial melting in the MTZ when other incompatible components (e.g. CO_2_, K_2_O and Na_2_O) are present. Since the subducted slab with sediments and serpentinites can be stagnant within the MTZ, it could release the above-mentioned components during dehydration and decarbonation to trigger partial melting in the MTZ much more easily than regions with no stagnant slabs. This has been evidenced by geophysical observations that partial melting at the top and bottom of the stagnant slab could take place in the MTZ beneath the western Pacific subduction zone [8]. In terms of carbon, both melting experiments and mineral inclusions in natural superdeep diamonds suggest that subducting carbonate-bearing oceanic crust will eventually undergo decarbonation melting in the MTZ, and much of the carbon therefore cannot be transported beyond 660 km, indicating that the MTZ is a principal barrier for deep carbon circulation and melting of recycled crust in the MTZ and could give rise to magmas with a ‘carbonate imprint’ that has characteristic metal stable isotopes such as low δ^26^Mg and high δ^66^Zn values [9]. Therefore, departing from conventional paradigms, the presence of C–H–O in the MTZ shifts the understanding of the origins of OIB-type intraplate magmatism, emphasizing the significance of upper mantle material recycling rather than rigid reliance on lower mantle circulation. Moreover, recent studies of superdeep diamond inclusions show that majoritic garnet presents a pronounced oxidation state increase with depth, attributable to the MTZ-derived carbonated fluids or melts acting as the oxidizing agents. The BMW system is thus a perfect laboratory to investigate the oxidation state variation as well as the speciation of deep mantle fluids, diamond formation and melting processes in the MTZ. Unlike traditional arc volcanism, dehydration-induced melting in the BMW, driven by MTZ-derived volatiles, complicates our understanding of subduction-related magmatism (Fig. 1). For instance, as a super-volcano, the formation of Changbai Mountain stands as a testament to the profound impact of the superimposition of volatiles derived from the MTZ on volcanic activity within the BMW and the Earth's surface processes. This complex influence extends to the subcontinental lithospheric mantle (SCLM), where MTZ-released volatiles are responsible for the thinning of lithosphere via a lowering of its strength and modification of its rheological property. Furthermore, the substantial introduction of volatiles into the BMW establishes a dynamic relationship with intraplate seismicity, prompting an exploration of the intricate links between volatile introduction and seismic activities.

In addition to C–H–O, other volatiles present in the MTZ, such as halogens, nitrogen, sulfur and noble gases, also play crucial roles. The halogens, owing to their subtly different chemical properties, impose constraints on a multitude of processes occurring in both the solid Earth and its surface reservoirs. The heavy halogen ratios observed in MORB and OIB exhibit a remarkable consistency (Br/Cl = (2.8 ± 0.8) × 10^−3^ and I/Cl = (60 ± 30) × 10^−6^), despite significant variations in halogen concentrations [10]. These ratios, unaffected by partial melting or fractional crystallization processes, offer a distinctive fingerprint for identifying specific halogen sources, such as seawater, altered oceanic crust (AOC) or sediments. These sources are transported from the surface to mantle at subduction zones, a process once believed to extend to depths of only ∼100 km. However, recent experimental studies have indicated that the MTZ might serve as a substantial deep reservoir for halogens, given their compatibility with the major mineral facies present in the MTZ. Nevertheless, despite these halogen solubility experiments on MTZ minerals, whether and to what extent halogens can be subducted in deep mantle is subject to debate, particularly for heavy halogens such as Cl, Br and I. A recent study estimated that up to 2% of the initial F, 50% of the initial Cl, 93% of the initial Br and 97% of the initial I entering subduction zones would be lost between the trench and eclogite stability field [11]. Even for F, a halogen well acknowledged to more readily enter the MTZ, there are opinions against this supposition and the suggestion that F would preferentially remain in the peridotite of the lowermost upper mantle instead of being introduced into the MTZ. In particular, the key halogen-carrier phases during subduction beyond sub-arc depth are still very poorly constrained, and how halogens behave during partial melting of the subducting slab in the deep mantle remains mysterious (Fig. 1). In addition, very little is known about the effect of halogens on melting relations in the mantle, as most previous experimental work has been centered on the crust level, rather than under MTZ conditions.

Likewise, our understanding of sulfur and nitrogen in the MTZ remains notably poor, despite their significance in sustaining life, ore deposit formation, volcanic eruptions and global climate change regulation. Limited by the inaccessibility of natural samples from the MTZ, most published data come from laboratory experiments that mainly focus on the solubility and solution mechanisms pertinent to characterizing their transport behaviors in silicate magmas. The relation between sulfur concentration at sulfide saturation (SCSS) and melt composition has been extensively investigated, particularly for peridotitic melt, and has increased our understanding of the fate of sulfur in a magma ocean [12]. However, all these studies, even under MTZ conditions, center mainly on the SCSS and relative timing of sulfide segregation during magma ocean crystallization. None of the work focuses on the behavior of sulfur during melting of the stagnant subducted slab and the storage of this vital volatile in the MTZ remains a significant unknown, largely impeding our understanding of the deep sulfur cycle. Furthermore, given the strong affinity of chalcophile elements, such as gold, silver and copper, with sulfide, these precious metals could form water-soluble sulfide complexes, and as the stagnant subducted slab in the MTZ slowly dehydrates, gold/silver/copper-rich fluid will be produced gradually, which might be of great significance for gold/silver/copper mineralization in the BMW system [13]. For nitrogen, its strong affinity with metallic Fe in the mantle makes Fe^0^ the major host of mantle N. If there is 0.1 wt% metallic Fe in the MTZ as suggested from previous work, N concentration would be 100–2000 ppm considering 0.1–2 wt% N is dissolved in Fe^0^. This indicates that the MTZ has great potential to store a substantial amount of N [14]. Solubility experiments further show that wadsleyite and ringwoodite can accommodate a minimum of 8.0 atmospheric masses of N [15]. However, the MTZ has often been overlooked when estimating the nitrogen cycle in the Earth. One reason is that our knowledge of N recycling during subduction is mostly limited to a relatively shallow level of the sublithospheric mantle (<150 km); another reason is that if we consider whole mantle circulation and oxidation state variation between the MTZ and the rest of the mantle, particularly in the BMW system where melting of stagnant subducted slabs occurs, the behavior of N is still very poorly constrained. More petrologic and experimental data of the MTZ are needed to justify the hitherto hypothetical MTZ reservoir for N, and to establish a complete N cycle of the Earth.

Compared with C–H–O–N–S and halogens, we know even less about the roles and behaviors of noble gases (He, Ar, Ne, Kr, Xe) in the MTZ. It appears to be more widely accepted that noble gases could be subducted to the mantle, facilitated by high solubility in amphibole as its mineralogical A-site is an energetically favorable position for noble gases. Additionally, many hydrous minerals in subducting slabs, such as serpentine and chlorite, share structural similarities with the A-site in amphibole, and this resemblance identifies these hydrous minerals as potential hosts for noble gases in deep mantle. It is thus extrapolated that if the subducting slab reaches the MTZ and becomes stagnant there, the MTZ could serve as a more substantial noble gas reservoir than previously thought. However, due to the scarcity of samples directly from the MTZ, this has never been verified. The most relevant advance was obtained by superdeep diamond fluid inclusions from the MTZ, which showed that primordial and recycled high ^3^He/^4^He signatures come from the deep mantle beneath a depth of 410 km, but not necessarily from the MTZ per se [16]. This work highlights that the MTZ serves as a significant heterogeneous reservoir accessed by rising plumes, ultimately giving rise to OIBs with recycled material signatures.

Apart from being introduced by slab subduction and stagnation, the volatiles in the MTZ could come from the lower mantle by large-scale mantle convection. Moreover, MORB units that are deeply subducted to the core mantle boundary (CMB) and subsequently entrained by upwelling plumes tend to accumulate in the MTZ due to the buoyancy effects of post-garnet phase transition, which might contribute to volatile accommodation in the MTZ even though these subducted slab remnants might have lost many of the volatiles during the long journey. Volatiles introduced by these processes could be expelled from the MTZ by younger subduction which might induce volatile-assisted melting above and below the MTZ, substantially affecting the BMW dynamics [7]. However, there has been limited research conducted on this volatile cycling mechanism ‘from bottom to top’ within the MTZ, highlighting a pressing need for further investigation. Subsequent research endeavors should prioritize the critical information from superdeep diamond inclusions and samples from the Earth's deep interior (such as OIBs) to help us decipher the mystery of hidden volatile cycling in the deep Earth, with a specific emphasis on the heterogeneous MTZ.

In summary, the MTZ is a much more significant volatile repository than previously thought. As a missing link in classic tectonic theory and total estimates of the volatile cycles of the Earth, the MTZ and its overlying BMW harbor many intriguing mysteries yet to be resolved and have great potential to fundamentally change our perspectives. Regions with BMW structure (e.g. western Pacific, eastern Australia, western Zealandia and southern South America) are excellent targets for such studies. A better understanding of volatile solubility in MTZ mineral phases and the release mechanism from these inventories is of great importance in confining the deep volatile cycle. In addition to the volatile solubility experiments, *ab initio* simulation, data obtained from natural diamond inclusions in the MTZ, the behavior of volatiles in the subducting slab from subduction to stagnation in the MTZ and their effects on deep crust–mantle metasomatism/interaction triggered by dehydration and decarbonation of the stagnant slab (Fig. 1), are particularly worthy of attention. Further development of micro-scale analytical techniques and more advanced volatile analysis methods is desperately needed to quantitatively constrain the volatile budget in deep Earth.
